# Validation and quantification of left ventricular function during exercise and free breathing from real-time cardiac magnetic resonance images

**DOI:** 10.1038/s41598-022-09366-8

**Published:** 2022-04-04

**Authors:** Jonathan Edlund, Kostas Haris, Ellen Ostenfeld, Marcus Carlsson, Einar Heiberg, Sebastian Johansson, Björn Östenson, Ning Jin, Anthony H. Aletras, Katarina Steding-Ehrenborg

**Affiliations:** 1 Clinical Physiology, Department of Clinical Sciences Lund, Lund University, Skåne University Hospital, Lund, Sweden; 2 Laboratory of Computing, Medical Informatics and Biomedical – Imaging Technologies, School of Medicine, Aristotle University of Thessaloniki, Thessaloniki, Greece; 3 Wallenberg Centre for Molecular Medicine, Lund University, Lund, Sweden; 4 Siemens Medical Solutions USA, Inc., Chicago, IL USA

**Keywords:** Magnetic resonance imaging, Cardiovascular diseases, Software

## Abstract

Exercise cardiovascular magnetic resonance (CMR) can unmask cardiac pathology not evident at rest. Real-time CMR in free breathing can be used, but respiratory motion may compromise quantification of left ventricular (LV) function. We aimed to develop and validate a post-processing algorithm that semi-automatically sorts real-time CMR images according to breathing to facilitate quantification of LV function in free breathing exercise. A semi-automatic algorithm utilizing manifold learning (Laplacian Eigenmaps) was developed for respiratory sorting. Feasibility was tested in eight healthy volunteers and eight patients who underwent ECG-gated and real-time CMR at rest. Additionally, volunteers performed exercise CMR at 60% of maximum heart rate. The algorithm was validated for exercise by comparing LV mass during exercise to rest. Respiratory sorting to end expiration and end inspiration (processing time 20 to 40 min) succeeded in all research participants. Bias ± SD for LV mass was 0 ± 5 g when comparing real-time CMR at rest, and 0 ± 7 g when comparing real-time CMR during exercise to ECG-gated at rest. This study presents a semi-automatic algorithm to retrospectively perform respiratory sorting in free breathing real-time CMR. This can facilitate implementation of exercise CMR with non-ECG-gated free breathing real-time imaging, without any additional physiological input.

## Introduction

Cardiovascular magnetic resonance (CMR) imaging typically uses an ECG-gated acquisition technique during breath-hold for assessment of cardiac volumes and function. Similarly, conventional flow measurements from phase-contrast (PC) CMR are also dependent on a stable ECG signal and a steady heart rate and are acquired during breath-hold or free breathing^1–3^. ECG signals are affected by body motion, especially during exercise, and especially at higher intensities^4^. Furthermore, adequate breath-hold is challenging during exercise and may induce a Valsalva-like maneuver affecting cardiac physiology^4^. These issues make CMR complicated for anything but breath-hold protocols at rest. These challenges can be overcome by brief periods of exercise cessation allowing for a stable ECG signal and breath-hold during acquisition, resuming exercise between acquisitions^5–9^. However, exercise cessation and breath-hold imaging are not truly representative of the physiological state of exercise.

Non-ECG-gated continuous real-time (RT) cine and PC CMR sequences can be used to overcome the limitations of inadequate ECG signals and non-breath-hold scenarios and they are recommended clinically when ECG-gated CMR is not possible^3,10–16^. However, the use of RT sequences comes with a cost of lower spatial or temporal resolution. Furthermore, when acquiring images during free breathing, respiration leads to cardiac translational motion within the thoracic cavity and introduces through-plane motion within the imaging plane. This leads to a risk of acquiring the same cardiac imaging slice multiple times. Moreover, respiration in itself has physiological effects on ventricular volumes^17–19^. Thus, volumetric quantification should ideally be performed in the same respiratory state to obtain non-confounded and accurate measurements.

Free breathing RT CMR has been validated for the quantification of ventricular volume and function using additional physiological data such as ECG registration or plethysmography during image acquisition^20–23^. However, these physiological data are not always available during exercise. To advance clinical implementation of exercise CMR, there is a need for a validated method to assess ventricular volume and function in RT CMR images without collecting additional registered physiological data.

The three aims of this study were first to develop a post-processing algorithm to assess ventricular volume and function from free breathing RT CMR with respiratory sorting. Second, to determine the image acquisition time needed for the algorithm to construct complete RT short-axis stacks. Third, we aimed to validate the accuracy of this algorithm in images acquired both at rest and during exercise.

## Methods

### Study design

Eight healthy volunteers (four female) and eight patients (three female) from the clinical workflow were prospectively included (Table 1) for CMR at rest. The only inclusion criterion for patients was normal sinus rhythm during image acquisition. All research participants were scanned on a 1.5 T MAGNETOM Aera (Siemens Healthcare, Erlangen, Germany), acquiring both ECG-gated and RT images at rest. In addition to CMR at rest, all eight healthy volunteers underwent exercise CMR with RT imaging.

**Table 1:** Participant characteristics. Cardiac volumes and mass at rest were determined from bSSFP cine imaging.

	Healthy volunteers (n = 8)	Patients (n = 8)
Age (years)	44 [38–53]	56 [51–59]
Female:male ratio	4:4	3:5
Height (cm)	175 [169–182]	175 [171–184]
Weight (kg)	68 [65–72]	78 [73–88]
BSA (m^2^)	1.83 [1.75–1.90]	1.96 [1.88–2.02]
**Rest**		
Heart rate, ECG-gated (beats/min)	59 [56–67]	66 [51–77]
Heart rate, RT (beats/min)	64 [61–71]	75 [65–80]
LV EDV, ECG-gated (ml)	155 [137–179]	167 [145–229]
LV ESV, ECG-gated (ml)	67 [53–84]	92 [71–104]
LV SV, ECG-gated (ml)	91 [82–99]	78 [69–102]
LVM, ECG-gated (g)	85 [69–104]	92 [77–123]
LVMI, ECG-gated (g/m^2^)	45 [39–56]	48 [41–63]
LVM, RT planimetry (g)	86 [74–102]	92 [84–116]
LVMI, RT planimetry (g/m^2^)	46 [41–55]	49 [43–59]
Cardiac Output, ECG-gated planimetry (l/min)	5.65 [4.88–6.26]	5.06 [4.66–6.83]
**Exercise**		
Heart rate, RT planimetry (beats/min)	125 [115–132]	
Heart rate, RT PC (beats/min)	129 [124–132]	
LV SV, RT planimetry end expiration (ml)	101 [96–112]	
LV SV, RT planimetry end inspiration (ml)	99 [95–109]	
LV SV, RT flow (ml)	94 [84–108]	
LVM, RT end expiration (g)	87 [72–95]	
LVM, RT end inspiration (g)	83 [70–94]	
Cardiac Output, RT planimetry end expiration (l/min)	12.47 [11.36–13.49]	
Cardiac Output, RT planimetry end inspiration (l/min)	12.66 [11.27–13.38]	
Cardiac Output, RT flow (l/min)	12.22 [11.39–13.26]	

Values presented as median [IQR].

*bSSFP* balanced steady state free precession, *BSA* body surface area, *ECG* electrocardiogram, *RT* real-time, *LV* left ventricle, *EDV* end diastolic volume, *ESV* end systolic volume, *SV* stroke volume, *LVM* left ventricular mass, *LVMI* left ventricular mass index, *PC* phase-contrast.

### Exercise protocol

Exercise was performed in the supine position using an MR-compatible cycle ergometer (Lode, Groningen, The Netherlands). Work rate started at 50 Watts for all research participants and was manually ramped up until a moderate intensity was reached, defined as a heart rate of approximately 60% of estimated maximum heart rate. Estimated maximum heart rate was calculated as 220 beats per minute minus participant age. Images were acquired while the participants continued to exercise at a fixed work rate.

### ECG-gated CMR

At rest, standard ECG-gated balanced steady-state free precession (bSSFP) sequences were used to acquire cardiac images in a short-axis stack, and in 2-chamber, 3-chamber, and 4-chamber long-axis views during breath-hold. Typical sequence parameters were: echo time 1.1 ms, repetition time 2.2 ms, flip angle 67°, acquired in-plane spatial resolution 1.9 × 2.0 mm, slice thickness 8 mm with no slice gap and temporal resolution 40 ms.

### Real-time CMR

Real-time CMR images were acquired using a product real-time steady-state free precession sequence. Short-axis images with whole-heart coverage, 2-chamber, 3-chamber, and 4-chamber long-axis views were acquired during free breathing and in the absence of ECG-triggering. One thousand time-frames at rest and 800 time-frames during exercise were acquired per slice. Typically, 14–17 slices were needed to cover the whole heart from base to apex. Fewer time-frames were acquired per slice during exercise than at rest to shorten image acquisition time and to lower the risk of fatigue.

Typical sequence parameters were: echo time 1.1 ms, repetition time 2.2 ms, flip angle 60°, acquired in-plane spatial resolution 1.9 × 2.8mm, slice thickness 10 mm with no slice gap. Parallel imaging (GRAPPA factor 3) and Partial Fourier (factor 5/8) acceleration were used to enable an acquired temporal resolution of 32–37 ms.

Depending on the chosen acquisition time, RT image acquisition can cover single or multiple cardiac and respiratory cycles. It cannot always be expected that both end diastole (ED) and end systole (ES) will coincide with an end respiratory state (end expiration or end inspiration) in one single respiratory cycle. Thus, non-ECG-gated acquisitions need to collect data during multiple respiratory cycles to increase the likelihood of ED and ES coinciding with end expiration or end inspiration. To ensure that data acquisition would cover several respiratory cycles, with a typical respiratory rate of 12 breaths per minute at rest, acquisition time was chosen conservatively to ~ 34 s (1000 time-frames).

### Respiratory sorting of RT short-axis image stacks

Real-time short-axis stacks comprising time-frames in both ED and ES with respiratory sorting were constructed during post-processing. This was done using the newly developed “respiratory module” in the software Segment (http://segment.heiberg.se) ^24^, presented below and available on request for research collaborations. The respiratory module used an algorithm to perform respiratory sorting of images semi-automatically, whereas time-frames in ED and ES were selected manually. This was by design to allow the user to review and control the entire stack construction process.

### Implementation and development of the semi-automatic respiratory sorting algorithm

**Figure 1:**
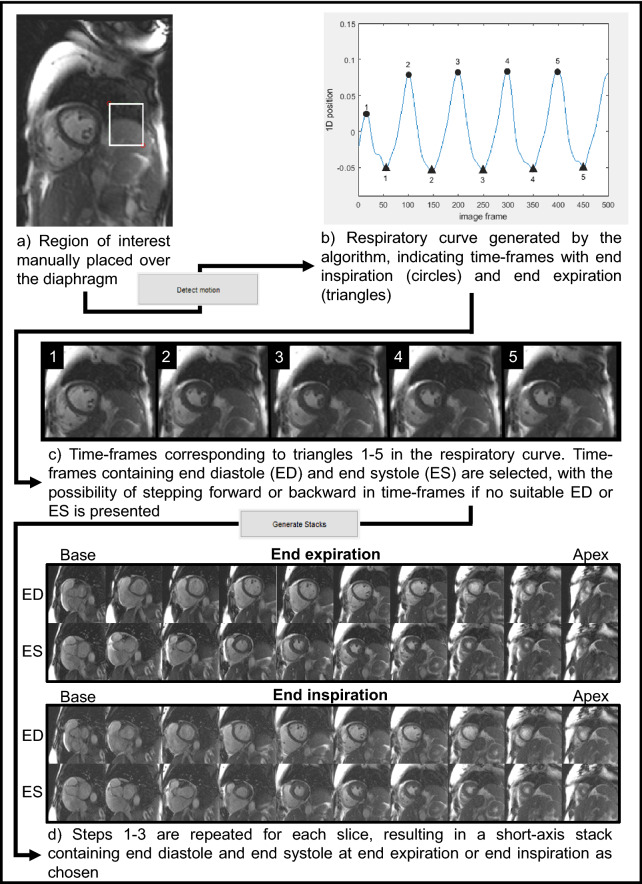
Flow chart for the respiratory module in Segment. The respiratory module in Segment utilized manual input to construct short-axis image stacks containing end diastole and end systole at end expiration or end inspiration from real-time CMR images. (**a**) A short-axis image with a respiratory region of interest (ROI, white box) placed over the superior border of the diaphragm. (**b**) The respiratory curve generated by the algorithm based on calculations from the placed ROI. Here the filled triangles annotate time-frames in which end expiration was identified, filled circles end inspiration. (**c**) An image gallery of the corresponding time-frames to the annotations in the respiratory curve (end expiration shown in the figure). These images were used for selecting time-frames in which end diastole and end systole coincide with end expiration. It was possible to manually step forward or backward several time-frames in each image if no suitable time-frames for end diastole or end systole were automatically suggested. While stepping forward or backward in time-frames the corresponding annotation moved in the respiratory curve, which allowed the user to ensure the current time-frame was still within end expiration or end inspiration. (**d**) The resulting short-axis image stacks containing end diastole and end systole at end expiration and end inspiration. *The software used for this figure was Segment 3.0 (*http://segment.heiberg.se*).*

The respiratory module generated a one-dimensional representation of the movement due to breathing (respiratory curve, Fig. 1) and performed respiratory sorting of the corresponding images via manifold learning (Laplacian Eigenmaps)^25^. The user placed a respiratory region of interest (ROI) covering the diaphragm (Fig. 1) and the algorithm created a 2D sequence from this cropped image region and applied the Laplacian Eigenmaps dimensionality reduction method for the 1D representation of the 2D sequence^26^.

We let the sub-images of the respiratory ROI be $x_{1} ,x_{2} , \ldots x_{N}$ where *N* is the number of time-frames of the cine. Given that the size of the ROI is approximately 40 × 40 pixels (for 128 × 128 cine frames), each sub-image $x_{i} , i = 1,2, \ldots ,N$ is a vector of 1600 dimensions representing a snapshot of the moving diaphragm. This image sequence was input to the Laplacian Eigenmaps nonlinear dimensionality reduction method which created a low-dimensional representation of the input data so that the distances between each point and its *k* nearest neighbors were minimized. In this way, temporally close sub-images were projected to similar values indirectly resulting in the discrimination of the end inspiration and end expiration sub-images. The locality of temporally close sub-images was captured by an adjacency graph with edges between neighboring nodes (sub-images) and edge-weights depending on the corresponding intensity similarity. Formally, it is required that the low-dimensional points $y_{1} ,y_{2} , \ldots y_{N}$ do minimize the following functional: $\varphi (y_{1} ,y_{2} , \ldots y_{N} ) = \mathop \sum \limits_{i,j} \|y_{i} - y_{j}^{2}\| W\left( {i,j} \right)$, where $W\left( {i,j} \right)$ are the weights of the adjacency graph $G$ of the points $x_{1} ,x_{2} , \ldots x_{N}$ computed using the Gaussian kernel:

$$W\left( {x_{i} ,x_{j} } \right) = \left\{ {\begin{array}{*{20}l} {exp\left( { - \frac{{\|x_{i} - x_{j}^{2}\| }}{{2\sigma^{2} }}} \right),} \hfill & {nodes\; i,\;j\;are\;connected\;in\;G } \hfill \\ {0,} \hfill & {otherwise,} \hfill \\ \end{array} } \right.$$

where $\left\| \cdot \right\|$ is the Euclidean norm and $\sigma^{2}$ is the variance of the Gaussian distribution, determining the influence of neighboring graph nodes (points). In our case, the nodes $i$ of the adjacency graph $G$ correspond to the images $x_{i}$ and each node is connected to its $k$ temporally nearest neighbors ($\left\lfloor {k/2} \right\rfloor$ before and $\left\lceil {k/2} \right\rceil$ after).

In this study, the low-dimension was selected to be one and therefore the resulting low-dimensional y-points were scalar values. In the resulting 1D representation, the extreme end points of the moving diaphragm (corresponding to end expiration and end inspiration) were projected to opposite positions and therefore their detection was reduced to local maxima and minima identification. As a result, two sets of images were produced, one containing end expiratory images and the other end inspiratory images (Fig. 1).

These image sets were presented in separate windows in the respiratory module, with each window displaying a time-frame at either end expiration or end inspiration as chosen by the user. The time-frame position of each window was annotated in the respiratory curve for each short-axis slice.

### User input and manual handling of RT short-axis image stack construction using the respiratory module

Two user inputs were needed for the algorithm:

1. The placement of the respiratory ROI for detection of respiratory cycle as described above. The resulting respiratory curve was created with the extreme points (maxima and minima) automatically defined to end expiration and end inspiration (Fig. 1).
2. The acceptance of the suggested ED and ES, or manually stepping forward and backward in time-frames in each slice to find maximum (ED) or minimum (ES) lumen blood volume. At rest, the duration of an end expiratory state in a research participant typically allowed for manual steps of ± 15 time-frames from the generated respiratory end points. For the end inspiratory state, which is a shorter interval than end expiration, manual steps of approximately ± 10 time-frames were considered. During exercise, where the respiratory cycle was shorter, manual steps of ± 8 time-frames were considered for end expiration and ± 5 time-frames for end inspiration.

### Rationale for manual input

By design, we chose the possibility of manual selection to allow for full control of all processing steps to ensure that the correct time-frames in ED and ES were chosen at the correct respiratory phase. The entire process, starting at the placement of the respiratory ROI, was repeated for each slice depicting the left ventricle in the RT short-axis image stack. Occasionally, when the diaphragm was not visible during the entire respiratory cycle, determination of end expiration or end inspiration was performed manually. This occurred mainly in the basal slices. Determination of respiratory states in these cases was performed by looking at the motion of the diaphragm outside of the ROI, the thoracic wall, and the greater lung vessels.

After short-axis image stack construction, all stacks were visually inspected within the Segment software to identify and remove potential duplicate slices which may have been included owing to cardiac translation during image acquisition. As a final check to avoid including too few or too many short-axis slices, left ventricular length at ED and ES was measured in the 4-chamber view and compared to left ventricular length in the short-axis images as calculated from slice thickness and number of delineated slices. If too many or too few short-axis slices were identified, either through visual inspection or differing left ventricular lengths in short-axis images compared to 4-chamber view, this was defined as a mismatch in left ventricular length.

Shorter image acquisition times than the original ~ 34 s per slice were simulated by cropping to the first ~ 17, 8, and 4 s of the acquired short-axis stack (corresponding to 500, 250 and 100 time-frames respectively).

### Real-time phase-contrast CMR

Real-time PC CMR images for flow measurement of the ascending aorta were acquired in healthy volunteers during exercise. A prototype sequence using segmented echo planar imaging was used with the following typical sequence parameters: repetition time 9.4 ms, echo time 4.9 ms, Echo-train-length 7, segments 4, flip angle 15°, reconstructed in-plane spatial resolution 3.1 × 3.1 mm, slice thickness 10 mm, VENC 200 cm/s, and reconstructed temporal resolution 38 ms with shared velocity encoding^27^.

Flow measurements in RT PC images were not matched for respiration. Thus, acquisition time did not need to ensure coverage of several respiratory and cardiac cycles, and an acquisition time of five seconds in free breathing was chosen.

### Image analysis

Image analysis was performed using Segment 3.0 (http://segment.heiberg.se^24,28^. The proposed methods were implemented as a module in the software. Assessment of left ventricular mass (LVM), end diastolic volume (EDV) and end systolic volume (ESV) was performed by manual delineation of the epicardial and endocardial borders of the left ventricle in the short-axis slices in both ECG-gated and RT images at ED and ES. Papillary muscles and trabecular tissue were excluded from the myocardial mass according to international recommendations^29^. Stroke volume (SV) was calculated as the difference between EDV and ESV, and ejection fraction (EF) as SV divided by EDV. Left ventricular mass was calculated as myocardial volume (the volumetric difference between epicardial and endocardial delineations) multiplied by 1.05 g/cm^3^ (the specific density of myocardium).

Stroke volume from PC CMR images during exercise was measured in the ascending aorta by using a semi-automatic vessel segmentation algorithm in Segment^28^. The net flow from the first diastole to the last diastole within a five-second acquisition window was divided by the number of heart beats to get an average stroke volume.

Heart rate was manually calculated for the RT cine images as no ECG-data were available. As heart rate may vary over time, especially during exercise, heart rate was calculated as the average from three slices per short-axis stack corresponding to near beginning, middle and end of acquisition.

### Intra- and inter-observer variability

Intra- and inter-observer variability of volumetric quantification was assessed in RT and ECG-gated images of eight healthy volunteers. Inter-observer testing of RT images included short-axis construction from a cropped acquisition time of ~ 17 s per slice (500 time-frames) using the respiratory module and manual delineation of the left ventricle. Intra-observer measurements were performed with more than one week between measurements. Inter-observer variability of the RT images was performed by a second observer. Both observers had > 1 year of CMR experience. Inter-observer variability of the ECG-gated images was performed by an observer with > 10 years of CMR experience.

### Statistical analysis

Continuous data are expressed as median and interquartile range [IQR]. Shapiro–Wilk tests were used to test for normal distribution. Bland–Altman analysis (bias ± standard deviation, SD) was used to compare differences between RT images and ECG-gated images, to compare SV between planimetry and flow quantification in RT images, and to evaluate intra- and inter-observer variability^30,31^. Intraclass correlation coefficient (ICC) was calculated with 95% confidence intervals using a two-way mixed model to assess the reliability of measurements of the same variable in RT image stacks constructed from differing acquisition times^32^. Coefficient of variation was calculated to assess precision in SV measurements during exercise. Pearson or Spearman’s rank correlation was used as appropriate for calculating correlation between measurements in RT images and ECG-gated images. A p-value of < 0.05 was considered statistically significant.

GraphPad Prism (version 8.4.1 for Windows, GraphPad Software, San Diego, California USA, www.graphpad.com) and IBM SPSS Statistics (version 26.0 for Windows, Armonk, NY: IBM Corp.) were used for statistical analyses.

### Primary measures for validation of the semi-automatic algorithm at rest and during exercise

The primary measures used for validation of the algorithm at rest were LVM, EDV, ESV and SV measured in RT short-axis images at end expiration in both patients and healthy volunteers. These were compared with a reference standard of ECG-gated images also acquired at rest and end expiration.

Reliability of LV volumetric and mass measurements between all completed RT short-axis stack constructions was tested using ICC to further evaluate necessary image acquisition time. For reliability during exercise, only LVM was compared to ECG-gated images as volumes, but not mass, were expected to differ between these two physiological conditions^17,33,34^. Similarly, RT CMR images analyzed at end inspiration were not used for volumes as ventricular volumes may differ depending on the respiratory state^17–19^.

Comparisons of LVM at end inspiration were included to test the feasibility of the algorithm to detect and construct complete short-axis stacks in both end respiratory states. Finally, comparisons of LVM measured from the first heart beat during image acquisition regardless of respiratory state was also included to gauge the impact of respiratory gating.

### Ethics approval and consent to participate

The Regional Ethical Review Board in Lund, Sweden, approved the study (reference number 948/2018). All participants gave written informed consent to participate in the study, including publication of data.

### Consent for publication

All participants gave written informed consent to publish data.

## Results

### Necessary acquisition time for complete construction of RT short-axis image stacks using the respiratory module

Matching end diastole (ED) and end systole (ES) to end expiration in all slices presented by the algorithm was possible in images with acquisition times of ~ 34, ~ 17 and ~ 8 s per slice (1000, 500 and 250 time-frames respectively). It was not possible to match ED and ES to end expiration in all slices when cropping to a ~  4 s image acquisition time (100 time-frames) in any of the research participants. Therefore, it was concluded that a ~ 4 s acquisition time was too short to allow for accurate volumetric quantification using the method described in the present study.

One case of mismatch in left ventricular length was found in one out of eight total RT short-axis stacks originating from the ~ 8 s acquisition time during exercise. This was due to time-frames containing ED and ES not coinciding with end expiration within the acquired images in the most basal slice of the ventricle. Thus, the most basal slice was not included in the resulting constructed short-axis stack. Note that measurements from this stack were still included in the volumetric results.

### Reliability of left ventricular (LV) volumetric and mass measurements for constructed RT image stacks

**Figure 2:**
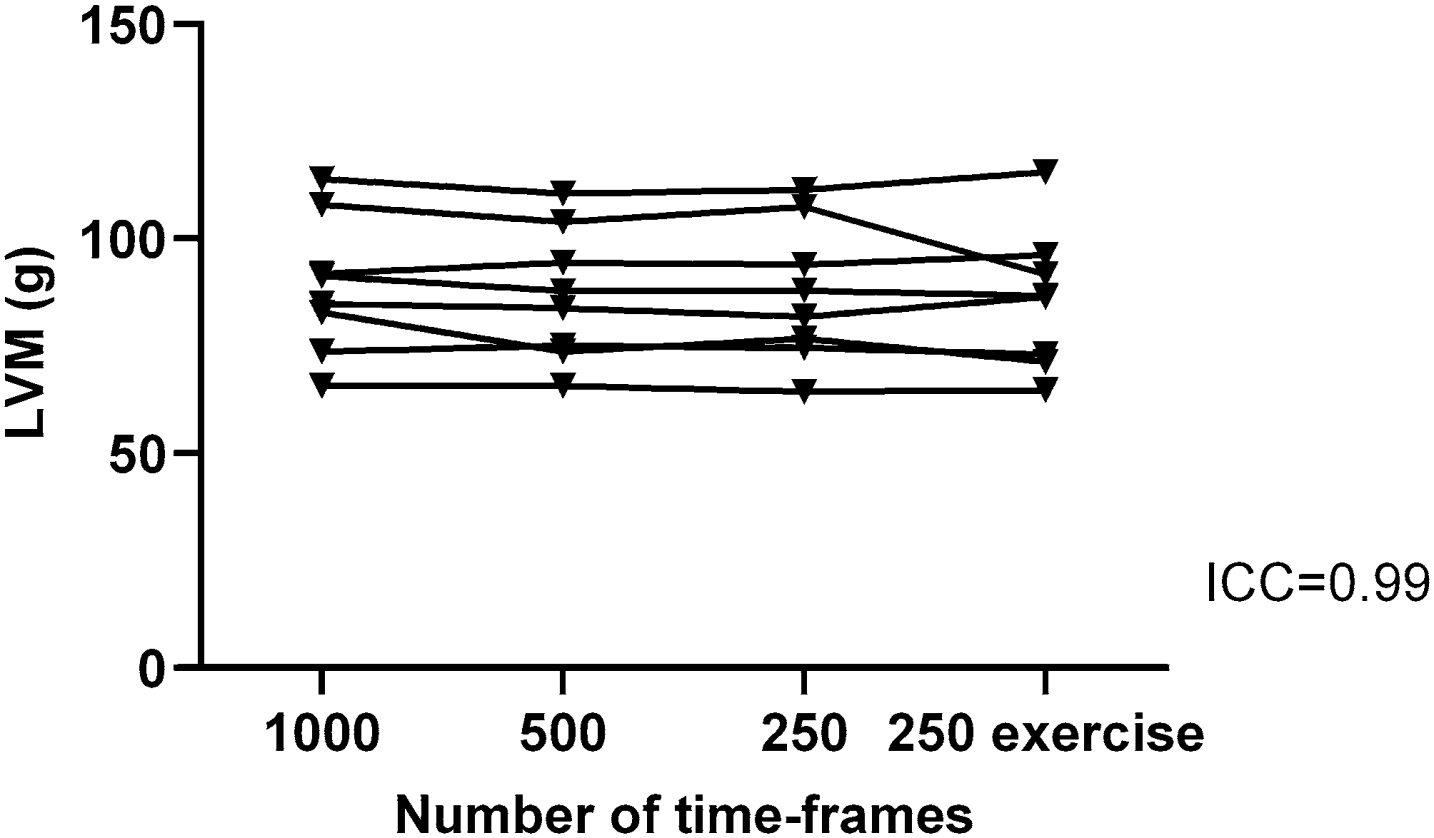
Reliability of left ventricular mass (LMV) measurements between real-time image stacks constructed from different numbers of time-frames at rest and during exercise in eight healthy volunteers. Reliability was high between measurements of left ventricular mass in real-time short-axis image stacks originating from 1000, 500, and 250 time-frames at end expiration. Real-time image stacks originating from 1000, 500 and 250 time-frames correspond to image acquisition times of ~ 34, ~ 17 and ~ 8 s respectively. The lines between the different time-frame measurements indicate the same research participants in the groups. 250 exercise shows measurements from images acquired during on-going exercise. *ICC* intraclass correlation coefficient.

Median values of LV volumes and mass derived from RT image stacks of 1000, 500, and 250 time-frames at rest as well as 250 time-frames during exercise are presented in Table 2 and Fig. 2. Reliability was high in LV volumetric and mass measurements in all constructed image stacks. ICC (95% CI) for 1000, 500 and 250 time-frames at rest and end expiration was for end diastolic volume (EDV) 0.994 (0.966–0.999), end systolic volume (ESV) 0.992 (0.974–0.998) and stroke volume (SV) 0.972 (0.900–0.994). ICC for all RT measurements (1000, 500 and 250 time-frames at rest and exercise, end expiration and end inspiration) was for LVM 0.990 (0.974–0.998).

**Table 2:** Median values of LV volume and mass measurements from RT short-axis image stacks originating from 1000, 500, and 250 time-frames at end expiration, end inspiration, regardless of respiratory state, at rest and during exercise in eight healthy volunteers.

		EDV (ml)	ESV (ml)	SV (ml)	LVM (g)	LVMI (g/m^2^)
Rest	1000 time-frames end expiration	160 [152–187]	70 [63–86]	95 [84–106]	88 [76–104]	47 [44–56]
	500 time-frames end expiration	158 [149–179]	71 [65–79]	89 [83–100]	86 [74–102]	46 [41–55]
	250 time-frames end expiration	161 [154–190]	72 [65–83]	92 [87–109]	85 [75–104]	45 [42–56]
	500 time-frames end inspiration	158 [144–185]	72 [61–80]	89 [82–104]	84 [71–100]	45 [39–54]
	First heart beat regardless of respiration	163 [151–187]	69 [57–83]	99 [88–107]	99 [79–116]	53 [45–62]
Exercise	250 time-frames end expiration	161 [152–184]	62 [53–73]	101 [96–112]	87 [72–95]	46 [39–53]
	250 time-frames end inspiration	163 [147–178]	66 [50–69]	99 [95–109]	83 [70–94]	45 [39–52]

Values are presented as median [IQR].

*RT* real-time, *EDV* end diastolic volume, *ESV* end systolic volume, *SV* stroke volume, *LVM* left ventricular mass, *LVMI* left ventricular mass index, *ICC* intraclass correlation coefficient, *CI* confidence interval.

### Agreement of LVM measurements in RT images versus ECG-gated images in healthy volunteers

**Figure 3:**
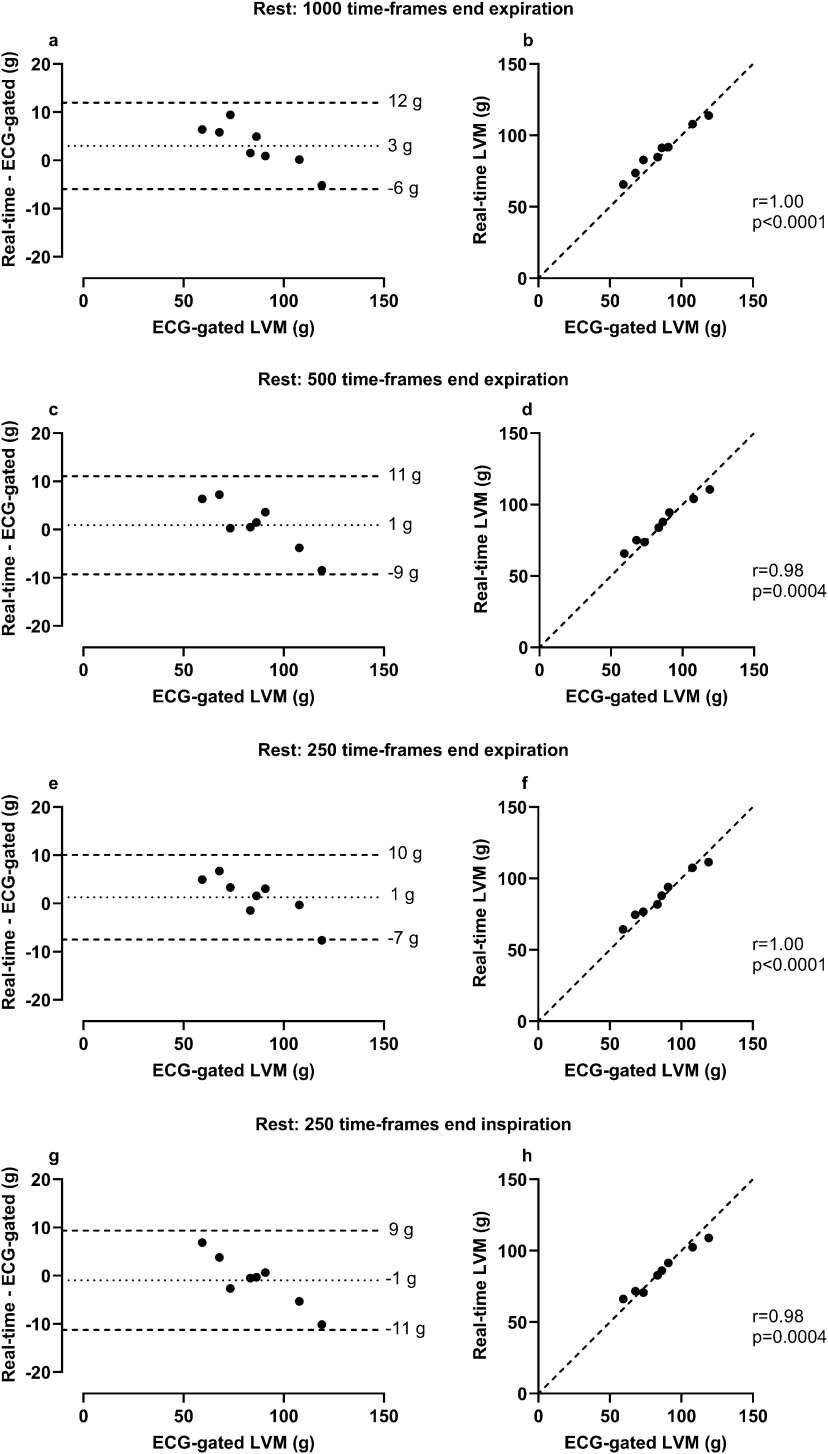

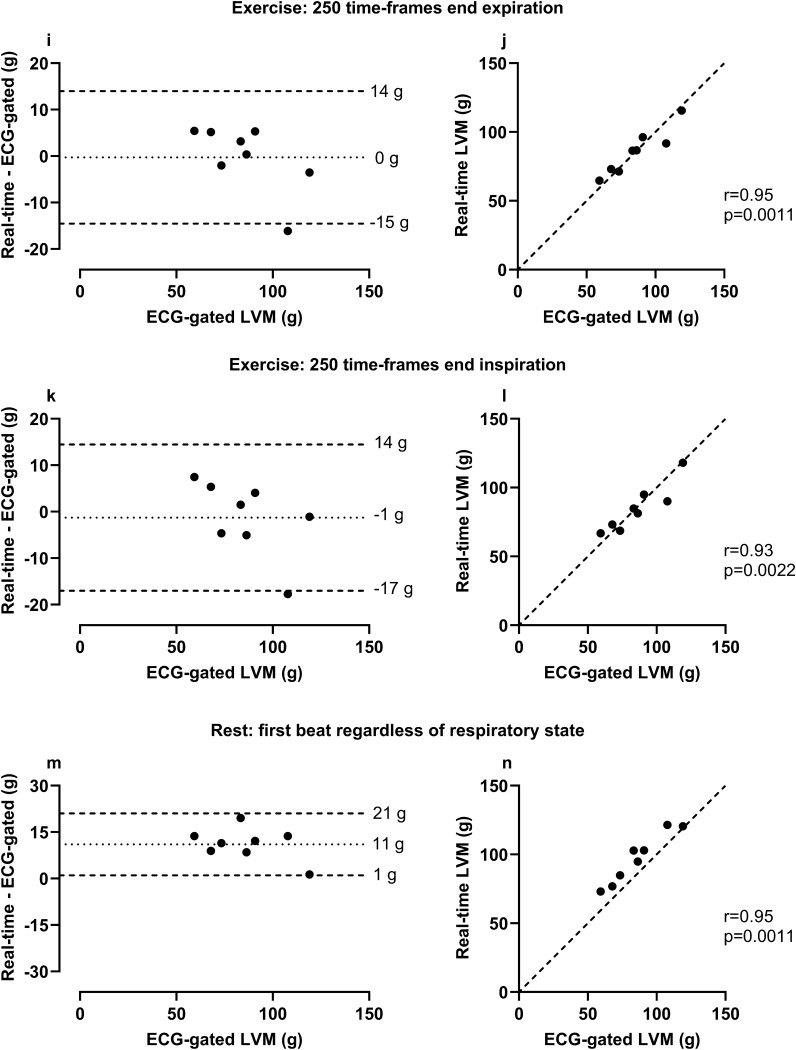
Agreement of left ventricular mass (LVM) measurements in real-time (RT) images versus ECG-gated images in healthy volunteers. Panels a–h show measurements made at rest, panels i–l during exercise and panels m–n regardless of respiratory state at rest. Note that for all panels, ECG-gated LVM was measured at rest. RT measurements were made at rest and end expiration unless otherwise specified**.** Bias was low and correlation high in all measurements in end-respiratory states, however one extreme value was seen in the two exercise measurements (i, k). In both cases, one basal slice was missing in one RT image stack during exercise. Bias was high in first heart beat measurements made regardless of respiratory state. *For Bland–Altman plots the dotted line represents bias, defined as the mean difference between real-time and ECG-gated measurements and the dashed lines represent the upper and lower 95% limits of agreement (bias* ± *1.96 SD of the difference). In the scatter plots the dashed line represents a line of identity.*

The bias of LVM in RT image stacks was for 1000 time-frames at rest at end expiration 3 ± 5 g, 500 time-frames at rest at end expiration 1 ± 5 g, 250 time-frames at rest at end expiration 1 ± 4 g, 250 time-frames at rest at end inspiration -1 ± 5 g, 500 time-frames during exercise at end expiration 0 ± 7 g, 500 time-frames during exercise at end inspiration -1 ± 8 g, and first heart beat regardless of respiratory state 11 ± 5 g compared with the reference of ECG-gated images at rest (see Fig. 3).

As measurements in 500 time-frames compared with 1000 time-frames showed high correlation, similar bias when compared to ECG-gated images for LVM and could be acquired with half the image acquisition time (~ 17 s), acquiring 500 time-frames was deemed more clinically relevant. Therefore, stacks constructed from 500 time-frames were chosen for comparison with ECG-gated images at rest as well as for intra- and inter-observer variability testing.

### Agreement of LV volumes and mass in RT images versus ECG-gated images in healthy volunteers and patients

**Figure 4:**
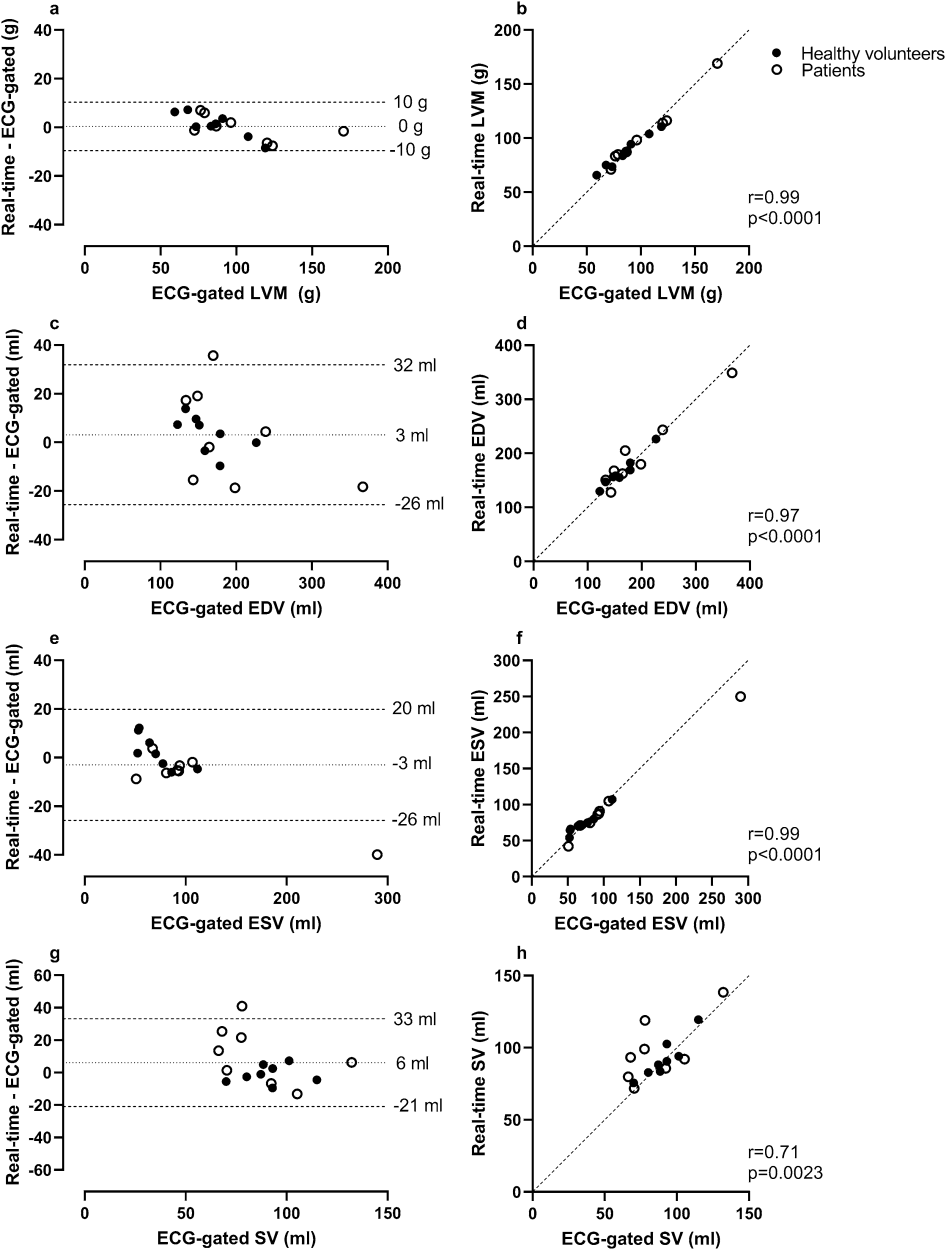

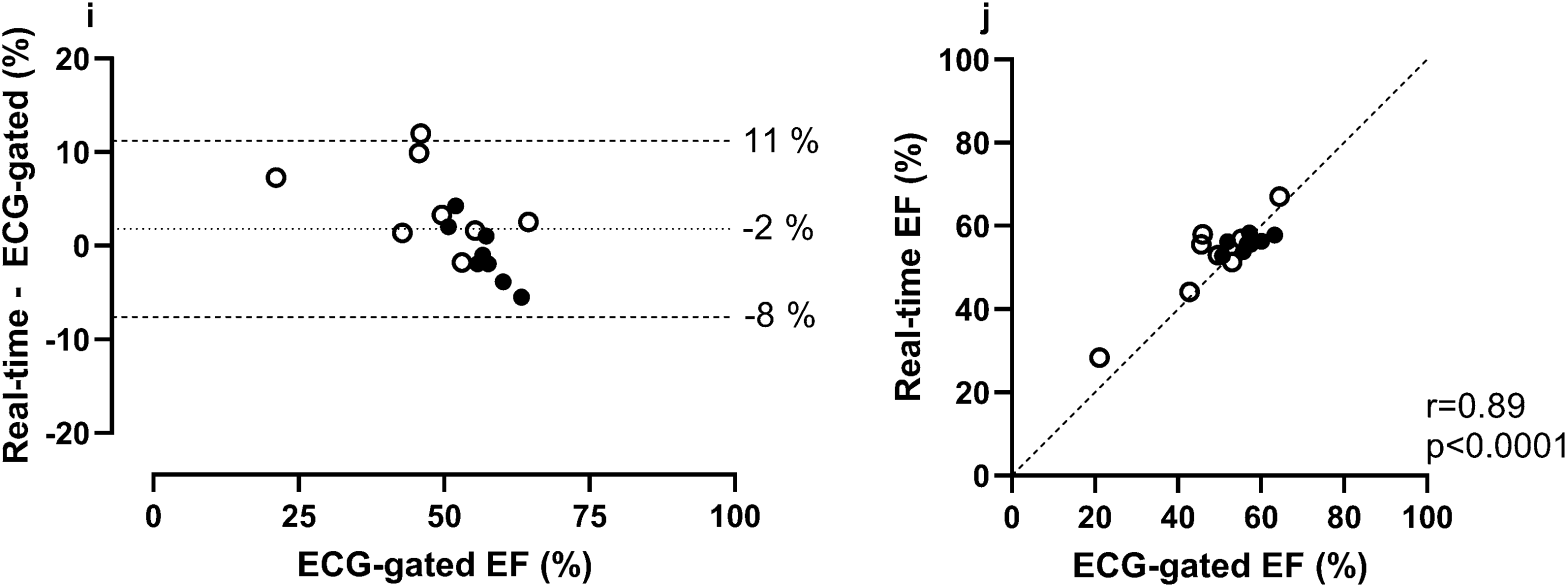
Agreement of left ventricular volumes, mass, and ejection fraction in real-time (RT) images at rest versus ECG-gated images at rest in eight healthy volunteers and eight patients. In all panels, RT measurements were made in stacks with a ~ 17 s image acquisition time per slice (500 time-frames). Bias was low and correlation high for all measurements. In the panels depicting EDV (c), SV (g) and EF (i), one point of measurement from the same patient can be seen outside the limits of agreement. In this case the ECG-gated images contained respiratory artifacts in several basal slices which may have impacted measurements. In the panel depicting ESV (e), one point of measurement from another patient can be seen outside the limits of agreement. In this case the patient had a severely dilated ventricle, making the absolute difference in measurements noticeable while the relative difference was comparable to other ESV measurements. *In the Bland–Altman plots the dotted line represents bias, defined as the mean difference between real-time and ECG-gated measurements and the dashed lines represent the upper and lower 95% limits of agreement (bias* ± *1.96 SD of the difference). In the scatter plots the dashed line represents a line of identity. LVM* = *left ventricular mass; EDV* = *end diastolic volume; ESV* = *end systolic volume; SV* = *stroke volume; EF* = *ejection fraction*.

The bias for LVM, EDV, ESV and SV between RT images at rest at end expiration originating from 500 time-frames compared to ECG-gated images at rest as a reference was 0 ± 5 g, 3 ± 15 ml, -3 ± 12 ml, and 6 ± 14 ml, respectively (Fig. 4).

### Validation of exercise RT planimetric stroke volume versus RT phase contrast stroke volume

**Figure 5:**
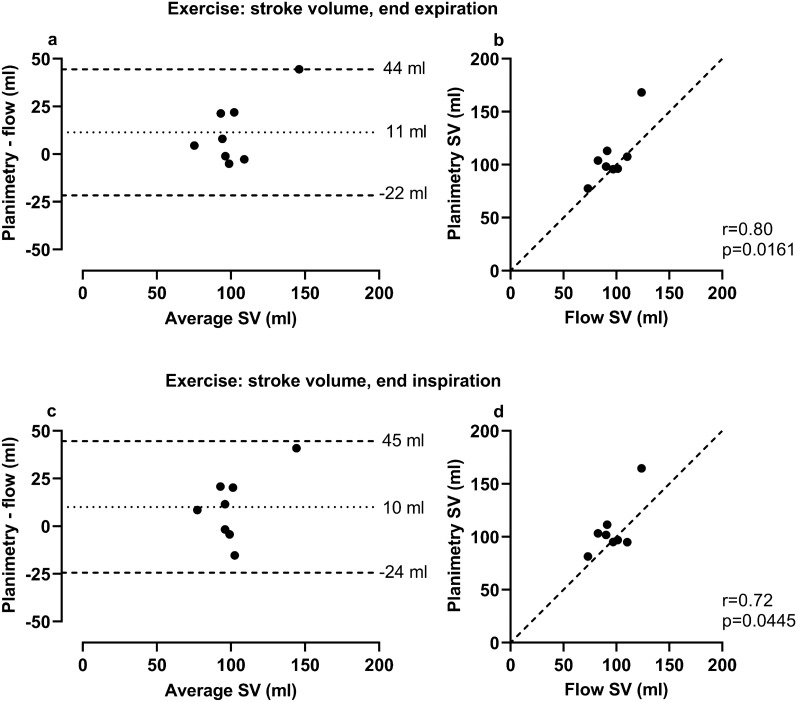
Comparison of stroke volume during exercise quantified by planimetry in real-time (RT) images at end expiration and end inspiration to flow in RT CMR images. Planimetric RT measurements were made in stacks with an ~ 8 s image acquisition time per slice (250 time-frames). Bias was low for left ventricular stroke volume (SV) when comparing planimetric measurements in RT CMR images with flow measurements in the ascending aorta in RT phase-contrast images in eight healthy volunteers. The outlier in each plot represents the same healthy volunteer in which stroke volume measured from flow was lower than from planimetry. No apparent irregularities in either modality were found in this individual. *In the Bland–Altman plots the dotted line represents bias, defined as the mean difference between planimetric and flow measurements, and the dashed lines represent the upper and lower 95% limits of agreement (bias* ± *1.96 SD). In the scatter plots the dashed line represents a line of identity.*

For left ventricular SV during exercise, mean difference between RT planimetric images and RT PC images as a reference standard was at end expiration 11 ± 17 ml (8.75% Coefficient of variation, CoV), and at end inspiration 10 ± 18 ml (10.15% CoV) (Table 1, Fig. 5).

### Agreement of LV volumes in RT images at end expiration versus end inspiration at rest and during exercise

The bias for EDV, ESV and SV between RT images at rest at end expiration compared to end inspiration was 0 ± 8 ml, 0 ± 4 ml and 0 ± 6 ml, respectively. During exercise, the bias for EDV, ESV and SV between end expiration compared to end inspiration was 3 ± 8 ml, 2 ± 7 ml and 1 ± 5 ml, respectively.

### Intra- and inter-observer variability

The intra- and inter-observer variability was excellent with low bias for EDV, ESV, SV and LVM in both RT images and ECG-gated images (Table 3).

**Table 3:** Intra- and inter-observer variability between RT planimetric and ECG-gated images. Intra- and inter-observer variability testing in real-time (RT) images included construction of RT short-axis stack originating from 500 time-frame stacks.

	RT images		ECG-gated images	
	Intra-observer	Inter-observer	Intra-observer	**Inter-observer**
LVM (g)	− 1 ± 4	8 ± 5	0 ± 6	6 ± 1
EDV (ml)	2 ± 3	3 ± 7	0 ± 4	− 4 ± 5
ESV (ml)	1 ± 3	− 3 ± 6	0 ± 3	− 2 ± 4
SV (ml)	1 ± 4	6 ± 4	0 ± 4	− 2 ± 6

Values are presented as bias ± SD.

*RT* real-time, *ECG* electrocardiogram, *LVM* left ventricular mass, *EDV* end diastolic volume, *ESV* end systolic volume, *SV* stroke volume.

## Discussion

We have developed and validated a new semi-automatic algorithm for respiratory sorting of non-ECG gated free breathing RT CMR to obtain accurate LV volumes and mass at rest and during exercise. The bias was low when comparing LVM measurements at rest with those during exercise, and the interobserver variability showed similar bias and variation (SD) when comparing RT with ECG-gated images, supporting the validity of the new algorithm.

Previous CMR studies as well as other imaging modalities have also used extraction of a respiratory curve or signal for proper volumetric measurement or to minimize imaging artifacts from respiratory motion ^35–39^. The novelty of the proposed method is the integration of an algorithm for a) extraction of a respiratory curve without any additional physiological data needed such as plethysmography from RT CMR short-axis images, b) construction of an image stack in the desired cardiac phase and respiratory state and c) ensuing analysis of the image stack. Thus, this study provides a validated post-processing method, and a so called “one-stop shop” for analysis, which may facilitate exercise CMR studies as all post-processing of exercise CMR non-ECG gated free breathing RT images can be done in the same software environment.

For complete RT short-axis stack construction, an image acquisition time of ~ 17 s per slice comprised adequate numbers of time-frames in which ED and ES occur during end expiration or end inspiration. However, in all but one out of eight cases, an acquisition time of only ~ 8 s during exercise also resulted in complete short-axis stacks. An acquisition time of anywhere between ~ 8 to ~ 17 s may therefore be sufficient. However, a shorter acquisition leads to an increased risk of a timewise mismatch of ED and ES, and respiration.

The low bias when comparing LV volumes between end expiration and end inspiration, both at rest and during exercise, are in line with previous results ^17^, suggesting that both end expiration or end inspiration may be used when quantifying LV volume and mass. Further, bias was somewhat high when comparing LV mass between RT images regardless of respiratory state to ECG-gated values. This underlines the importance of performing analysis in a predefined respiratory state, i.e. either end expiration or end inspiration, to minimize cardiac translation and through-plane motion. For the right ventricle, however, respiratory phase is of physiological importance as shown by previous studies indicating a more pronounced difference in right ventricular volumes between end expiration and end inspiration^17,40^.

The strength of the method described in the present study is the ability to ensure that all images analyzed are in the same respiratory phase, for example end expiration or end inspiration, and exercise can be continuously performed during the data acquisition, providing new insights to pump physiology during exercise. The feasibility of acquiring RT CMR during exercise and free breathing to assess volumes has previously been shown by Lurz et al*.*^20,22^. However, in contrast to the present study, Lurz et al. did not adjust for breathing in the post-processing analysis or during image analysis. This limits the evaluation of volumes due to cardiac translation and variation in ventricular filling caused by respiration during image acquisition^17–19^.

Results from the present study regarding good agreement between RT images and ECG-gated images are in concordance with La Gerche et al*.*^23^ and Claessen et al*.*^17^ who used RT CMR to quantify biventricular volumes both at rest and during exercise. There is a difference between these studies and the present, however, regarding the methodology of data acquisition. The studies by La Gerche et al. and by Claessen et al. used plethysmography to track and register a respiratory phase curve in real-time during image acquisition. An acquisition time of ~ 3.8 s per slice (100 time-frames) at rest was possible by acquiring sufficient frame repetitions to include at least one respiratory cycle per slice ^17^. The present study suggests a simpler data acquisition method of RT images, but at the cost of comparatively longer image acquisition times. Further, already acquired RT images in which plethysmography was not used can likely be analyzed in a desired end respiratory state using the proposed method, making it possible to include retrospective data as well.

Exercise CMR is a future tool to study physiological adaptations to exercise both in clinical and research settings. Several studies have either applied exercise cessation or breath-hold, or both, to obtain images of good quality^5–7,41,42^. The present study shows that RT CMR with free breathing combined with a post-processing algorithm to identify end expiration can be used to accurately measure volumes at rest, as well as mass at rest and during exercise. This allows for quantification of LV function in a setting which more closely represents the physiological state of on-going exercise. Another potential use of the algorithm is the ability to detect the respiratory phase. Thereby, it is possible to measure volumes during different parts of the respiratory cycle, and not just exclusively at end expiration as is typically the case in ECG-gated CMR.

Two statistical outliers were seen in the patient group when comparing volumes from ECG-gated images and RT images (Fig. 3). This could result from the presence of respiratory motion artifacts due to intolerance to breath-hold during image acquisition, making exact delineation of endocardial and epicardial borders more difficult. One could speculate that RT images in these cases may provide more accurate LV volumes as they do not require breath-hold and thus do not contain respiratory motion artifacts. Further, one of the outliers represented a patient with a severely dilated left ventricle. In this case, the absolute difference in measurements made the differences more pronounced when viewed together with the rest of the group, while the relative differences were on a similar level. As previously mentioned, one basal slice was missing in one RT image stack during exercise. This resulted in an outlier when comparing LVM between rest and exercise in the healthy controls, both when measured at end expiration and end inspiration. Finally, an outlier was seen when comparing planimetric stroke volume in RT images to RT aortic flow during exercise, both at end expiration and end inspiration. In this case, no apparent irregularities in either imaging modality were found to explain the differences.

Some limitations to the present study are to be noted. First, selecting the frames to construct a complete RT short-axis stack typically took 20 to 40 min, which may be considered by some to be labor intensive. However, when compared to manually inspecting 1000 time frames per slice for 14 to 17 slices to identify ED and ES within a chosen end respiratory state, processing time was considerably shortened. Additionally, the benefit of accounting for actual physiology in RT images, when ECG-gated acquisition is not possible, may outweigh the added analysis time. While additional processing power and software optimization would reduce post-processing time, the major time-consuming factor was dependent on manual user input. This was by design, in order to keep full control of which image slices were added to the image stack. However, automation of RT short-axis stack construction through new machine learning techniques may potentially decrease total analysis time by a substantial margin.

Only healthy volunteers underwent exercise CMR in the present study. However, by including measurements from cardiac patients at rest, we demonstrated clinical-setting feasibility and provided a more likely scenario of how the described method and algorithm would perform in the clinic.

The constructed RT image stacks contained only images at ED and ES and not an entire cardiac cycle per slice. In time-resolved ECG-gated images, time-frames adjacent to ED and ES can be helpful to differentiate papillary muscles and trabeculation to the endocardial border. As such, it could be more difficult to differentiate these structures in RT images. However, this risk was minimized by comparison to adjacent slices in the apical or basal direction, and there was good agreement between volumes and mass obtained from RT and those obtained from ECG-gated images.

Determination of ED and ES for the short-axis stacks was not performed identically in RT images and ECG-gated images. In ECG-gated images, one time-frame of the cardiac cycle was determined as ED and ES respectively for the entire short-axis stack. In the RT images, determination of ED and ES was performed on a per-slice basis as the cardiac cycle was not synchronized between slices due to a lack of ECG-gating. As the ventricle does not always contract in a perfectly synchronized pattern along the long axis, such as in patients with left bundle branch block or after myocardial infarction^43,44^, this could lead to discrepancies in what is considered ED or ES at different levels of the ventricle. However, this is likely not a major limitation in the current study population but may be of importance when applying this algorithm in cases with ventricular dyssynchrony.

Additionally, during exercise, the height of the research participants and the bore diameter did not always permit optimal table positioning for the ascending aorta to be at the scanner isocenter. While the feasibility of using RT PC during exercise in free breathing has been previously presented, isocenter positioning was not addressed^45–47^. Thus, it is unknown to what extent this affects flow measurements in the present study.

Last, we found a bias in stroke volumes during exercise between flow measurements and planimetric measurements in RT CMR. This may in part be explained by planimetric measurements not accounting for mitral regurgitation when assessing left ventricular stroke volume. On the other hand, the size of the bias implies that a potential mitral regurgitation is to be considered small. Furthermore, aortic flow does not include coronary blood flow proximal of the PC imaging plane in the calculated stroke volume; however, this can explain only a small part of the difference owing to the small proportion of coronary flow. Aside from strictly methodological differences, the bias could also result from RT flow images and short-axis images not being acquired simultaneously. While they were acquired during the same exercise session, small physiological differences owing to continuous on-going exercise could affect the left ventricular stroke volumes between the two image acquisitions. Further, the RT PC images had relatively low spatial and temporal resolution when compared to typical ECG-gated PC images recommended for clinical routine^10^. While the imaging parameters were mostly within recommended limits, they could nonetheless have added to the measured bias. These potential explanations for the differences between the two methods of measuring stroke volume further strengthen the argument that the reliability of RT planimetric measurements for volumetric quantification during exercise is high.

## Conclusion

This study presents a semi-automatic algorithm with retrospective respiratory sorting for assessment of left ventricular mass, volumes, and function from free breathing real-time CMR at rest and during exercise. The algorithm requires no ECG or additional physiological data during image acquisition. By acquiring images during ~ 17 s per slice (500 time-frames), complete construction of RT short-axis stacks was possible in all cases with a total post-processing time of typically 20 to 40 min. The validation shows good agreement and high correlation between measurements at rest and during exercise. As such, we present a new validated tool applicable to ventricular functional assessment of non-ECG triggered cine images acquired during free breathing as well as exercise.

## Supplementary Information

Supplementary Legends.

Supplementary Video S1.

## Data Availability

The datasets used during the current study are available from the corresponding author on reasonable request.
