# Supplementary material for: Validation and quantification of left ventricular function during exercise and free breathing from real-time cardiac magnetic resonance images
Source: Sci Rep. 2022 Apr 4;12:5611. doi: 10.1038/s41598-022-09366-8 (PMC8979972; doi:10.1038/s41598-022-09366-8)
Supplement: Supplementary file 1 — Supplementary Legends. [file 41598_2022_9366_MOESM1_ESM.docx]

**Supplementary figure 1**

The left panel shows cine short-axis images from ECG-gated CMR at rest (heart rate 67 beats per minute), middle panel shows real-time CMR at rest (heart rate 70 beats per minute), and to the right real-time CMR during exercise (heart rate 131 beats per minute) in the same healthy volunteer.
